# Supplementary material for: Deep neural network inference on an integrated, reconfigurable photonic tensor processor
Source: Nat Commun. 2026 Apr 9;17:3396. doi: 10.1038/s41467-026-71599-2 (PMC13066431; doi:10.1038/s41467-026-71599-2)
Supplement: Supplementary file 1 — Supplementary Information [file 41467_2026_71599_MOESM1_ESM.pdf]

Supplementary Information for

Deep Neural Network Inference on an Integrated, Reconfigurable Photonic Tensor Processor

Lennart Meyer[1], Jelle Dijkstra[1], Simon Tebeck[1], Liam McRae[1], Niklas Bahr[1,2], Daniel Steinmeyer[2], Sergey Koptyaev[3], Johana Bernasconi[3], Nikolay G. Pavlov[3], Maxim Karpov[3], John D. Jost[3], Wolfram Pernice[1], Frank Brückerhoff-Plückelmann[1]

[1]University of Heidelberg, Kirchhoff-Institut für Physik, 69120 Heidelberg, Germany

[2]Volkswagen AG, Berliner Ring 2, 38440 Wolfsburg, Germany

[3]Enlightra, Rue de Lausanne 64, 1020 Renens, Switzerland

Supplementary Note 1. Self-injection locking microcombs (SIL)

The traditional scheme for microcomb generation requires the use of tunable narrow-linewidth lasers as pump sources [1]. Such lasers are bulky and prevent the compact integration of soliton microcombs. Another way to generate frequency combs in chip-scale optical microresonators has recently been demonstrated using compact semiconductor lasers and the self-injection locking (SIL) effect [2-6]. The self-injection locking effect is based on light, which is reflected from surface inhomogeneities of the microresonator (Rayleigh back-scattering) [7-9] and directed back to the semiconductor laser diode. This forces the laser to be locked close to the microresonator resonance frequency, suppressing the laser noise while at the same time stabilizing the generation of microcombs.

Optical packaging:

The challenge in terms of optical packaging is the precise alignment of the laser diode to the Si_3N_4 chip (with <0.5µm accuracy), satisfying at the same time the optimal phase for SIL. The Supplementary Fig.1 illustrates the photos of a proof-of-concept assembly (left) as well as the packaged microcomb source (right).

The Si_3N_4 microresonator with a 485 GHz free spectral range (FSR) was pumped with a hybrid integrated distributed feedback (DFB) semiconductor diode laser to generate the microcombs

in SIL. The detailed SIL theory, early experiments are presented in the papers[5-9]. The optical subsystem consists of a ~100 mW DFB laser diode butt-coupled to the Si_3N_4 photonic chip input facet and a single-mode PM fiber pigtail at the opposite facet (Supplementary Fig.1). This subsystem allows the generation of a low noise soliton microcomb in the SIL. The precise integration between the DFB diode, Si_3N_4 chip, and output optical fiber was done with the precision alignment of all elements satisfying the stringent conditions for SIL [7], such as low loss and optical feedback phase control [8,9]. The single-mode PM FC/APC fiber array was glued to the chip facet for coupling output light from the photonic chip. A custom package (66 x 32 x 15 mm) featuring a thermoelectric cooler (TEC) element as well as a thermistor for temperature control is used to further isolate the optical subsystem and interface the laser diode and TEC, and thermistor with the electronic subsystem.

The performance of the packaged device is shown in Supplementary Fig.2(a)-(b). Supplementary Fig.2(a) represents the low noise single soliton microcomb spectrum with ~485 GHz spacing. Supplementary Fig.2(b) shows the frequency noise performance for the laser diode self-injection-locked to a high-Q Si_3N_4 microresonator. The blue curve is the free-running DFB laser diode, the red curve is the SIL laser diode (central line). The SIL LD achieves two orders of magnitude linewidth suppression at high frequency offsets (>10kHz) and is limited by thermal-refractive noise in the microresonator (represented by the black dashed line [10]). The Lorentzian linewidth for the comb's teeth is around 2 kHz. The spikes on the trace are artefacts originating from the measurement setup.

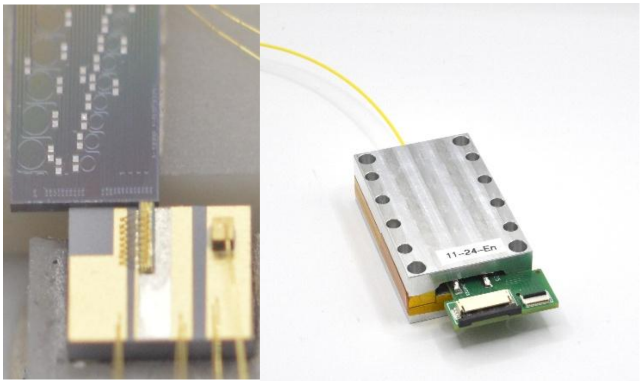

Supplementary Figure 1: Optical packaging of the microcomb. Left: LD is butt-coupled to the Si3N4 chip from one side and output pigtailed fiber from another side. **Right:** Generic packaged with SIL microcombs features TEC element and thermistor.

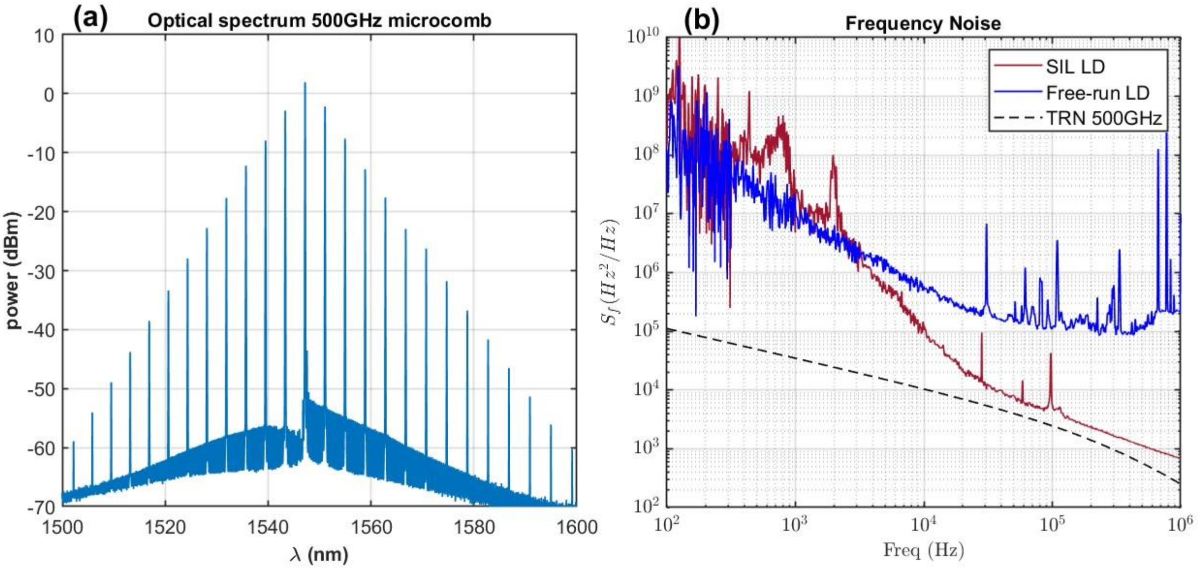

Supplementary Figure 2: Characterisation of the microcomb. (a), Optical spectrum of 500 GHz SIL microcomb. **(b),** Frequency noise suppression of SIL laser: blue curve – free run DFB laser, red curve – SIL, black dashed curve is thermorefractive noise for 500 GHz Si_3N_4.

Supplementary Note 2. Crossbar Structure

We encode each input vector element $(x_1, \ldots, x_M)$ onto a different wavelength channel using the on-chip modulators. The matrix is implemented as a waveguide crossbar array equipped with directional couplers that evenly distribute the optical power to all EAM cells. For an $M \times N$matrix (Supplementary Fig. 3), the horizontal couplers have splitting ratios of $1/(N -$

$j+1$) for column index j, and the vertical couplers have splitting ratios of $1/i$ for row index i. The matrix elements themselves are encoded in the EAM transmission. A frequency comb with a line spacing larger than the electrical bandwidth ensures interference-free detection of the summed optical intensities along the individual columns. The output power at each column photodiode corresponds to the inner product between the input vector and the respective weight.

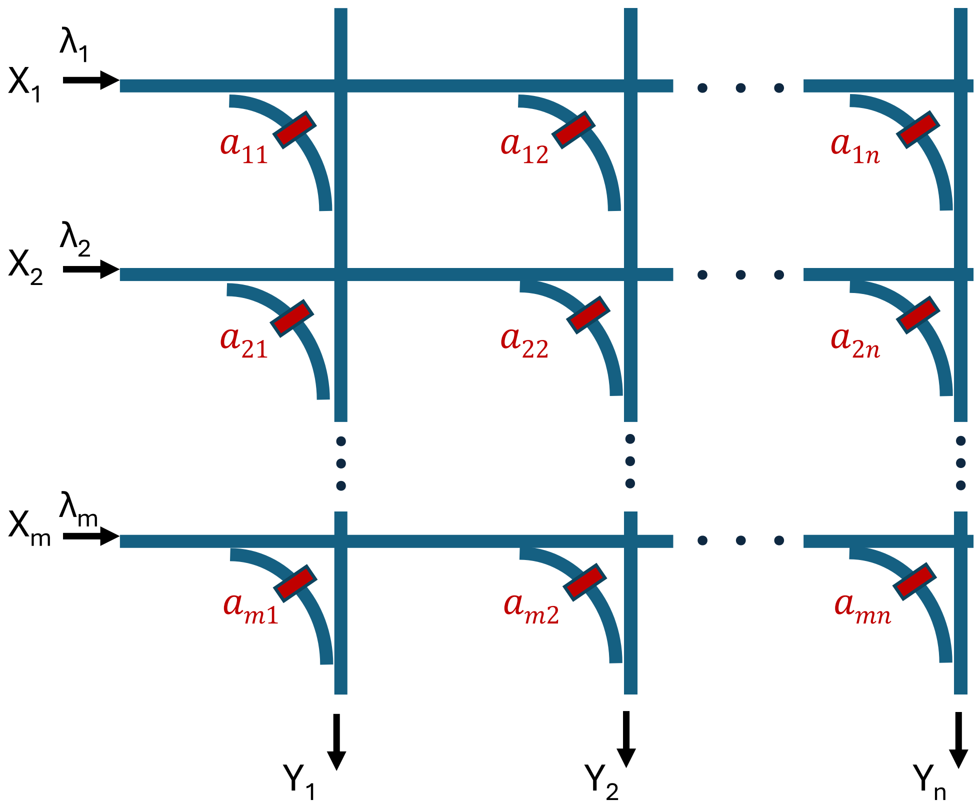

Supplementary Figure 3: Crossbar structure of the Photonic Tensor Core. We encode the input vector elements $x_1, \ldots, x_m$ onto different wavelengths carriers $\lambda_1, \ldots, \lambda_m$ and the matrix weights into each cell's EAM $a_{11}, \ldots, \mathrm{a}_{mn}$. The intensities at the end of each row $y_1, \ldots, y_n$ are proportional to the inner product of the input vector and the respective weights.

Supplementary Note 3. Tiling and Accuracy

The lower accuracy observed for CIFAR-10 is consistent with the substantially larger effective matrix sizes required by the network, which increase the severity of tiling in the present 9 input

3 output photonic tensor processor. In the CIFAR 10 model, the largest convolutional operation is the final 3 × 3 convolution with 64 input channels, corresponding to an effective input vector length of 3 × 3 × 64 = 576 per output channel, whereas the final classifier operates on the flattened 8 × 8 × 128 feature tensor, corresponding to an input vector length of 8 × 8 × 128 = 8192. This increase in vector dimension requires a much larger number of tiled partial MVMs and therefore a larger number of digital accumulation steps. While predominantly stochastic errors are expected to preserve approximately constant relative error under tiling, correlated or non-stochastic error components accumulate more strongly with the number of tiles and can limit performance at large matrix sizes. To isolate the contribution of the classifier tiling, we performed a hybrid inference experiment in which all layers up to and including the layer preceding the classifier were executed on the photonic hardware using experimentally measured activations, while the final fully connected layer was computed digitally. This increased the CIFAR-10 accuracy from 72% to 78%. Computing both the final 3 × 3 convolution (64 to 128 channels) and the fully connected layer were computed digitally, only yielded an addition 3% in accuracy up to 81%. These results support the conclusion that the extensive tiling required by the final fully connected layer is a dominant factor in the observed accuracy degradation, and that reducing non stochastic error components and increasing the native tensor core dimension are important directions for scaling to larger models

Supplementary Note 4. Bit Precision and MVM Error

To separate the contribution of the input output interface from the intrinsic tensor core error, we characterized the end-to-end transmission path from DAC through EAM and photodetection to ADC. Using random input data and measuring the digitized output of the ADC, we quantified the transmission error as a function of averaging and modulation voltage swing. Supplementary Fig. 4 shows that, at low averaging, the transmission error is small for large modulation swing, whereas at high averaging a systematic floor becomes visible. The noise floor increases for

larger voltage swings. This behaviour is consistent with residual EAM transfer function nonlinearity, which becomes apparent once stochastic noise is reduced by averaging. For the 1.0 Vpp setting, we extract a systematic transmission error of 0.3 %, which is small compared to the systematic error observed for full MVM execution. This indicates that the overall MVM error is not primarily limited by the electro-optic interface, but instead by the tensor operation itself, including weight programming and crossbar level distortions. Since the DACs and ADCs provide 14 bit resolution and the measured transmission error is low, we model the activation path with an effective input precision on the order of 8 bits for the quantization comparison.

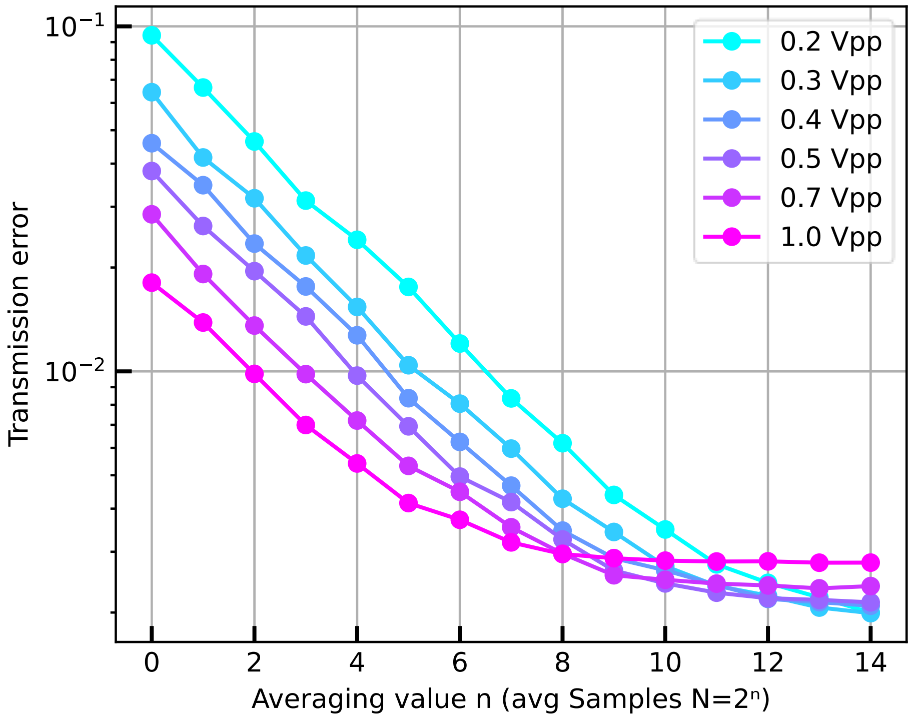

below.

Supplementary Figure 4: Transmission Error vs Averaging value. Transmission measurement of the DAC-EAM-PD-ADC Path for different Volt-Peak-Peak configurations of the modulation Voltage, showinga difference in non-linearity for averaging settings

Building on this, we compare the measured photonic MVM error to an ideal digital reference in which inputs are quantized to 8 bits and weights are quantized to different bit widths (Supplementary Fig. 5). In this comparison, the single shot low latency operating point with

MVM error (19.4 ± 0.5) % is closest to a digital MVM with approximately 3 bit weights, while the four shot averaged precision mode with MVM error (10.9 ± 0.3) % is closest to approximately 4 bit weights. While the output error can in principle arise from both weight and input errors, the small systematic error of the DAC-EAM-PD-ADC path implies that the dominant contribution in our experiments is associated with the weight programmed tensor operation rather than an errorless input assumption.

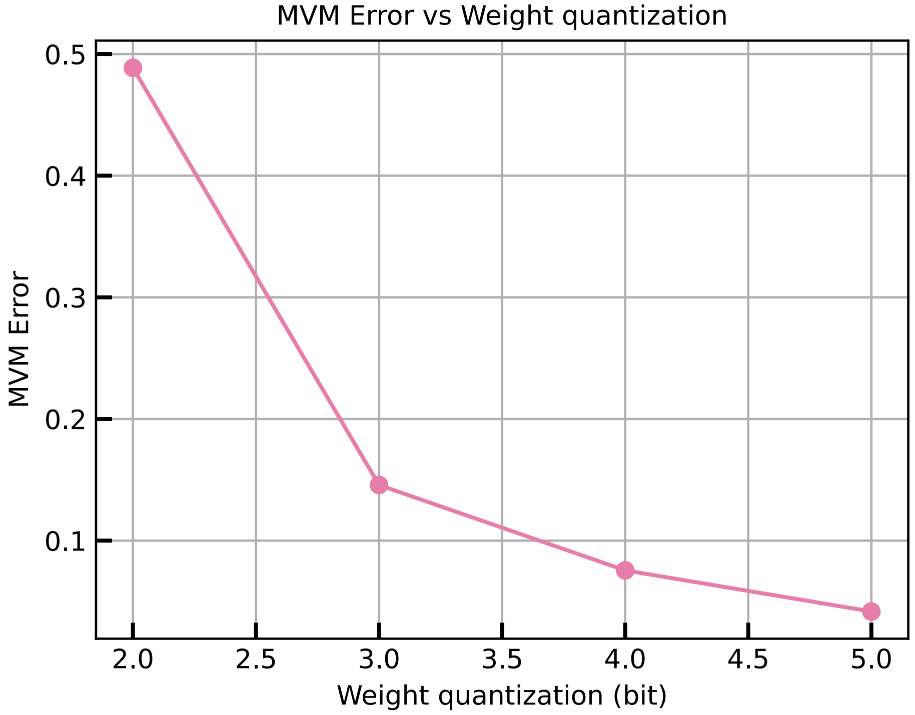

Supplementary Figure 5: MVM Error vs Weight quantization. Calculated MVM error for a digital system with inputs to quantised to 8 bits and different weight quantization

Supplementary Note 5. EAM Characterisation

To assess the modulation bandwidth headroom, we performed a high-speed characterization of the electro absorption modulators. We generated an 8 GBaud electrical drive sequence and recorded the corresponding optical output using a high bandwidth photo receiver, connected to an oscilloscope from which we obtained the eye diagram shown in Supplementary Fig. 6. The

open eye confirms that the EAMs support symbol rates beyond the 1 GHz per second operating point used for inference. In the present PTP, the system level input/output rate is therefore limited by the DAC and ADC sampling rates and their efficiency trade-offs, rather than by the intrinsic EAM bandwidth.

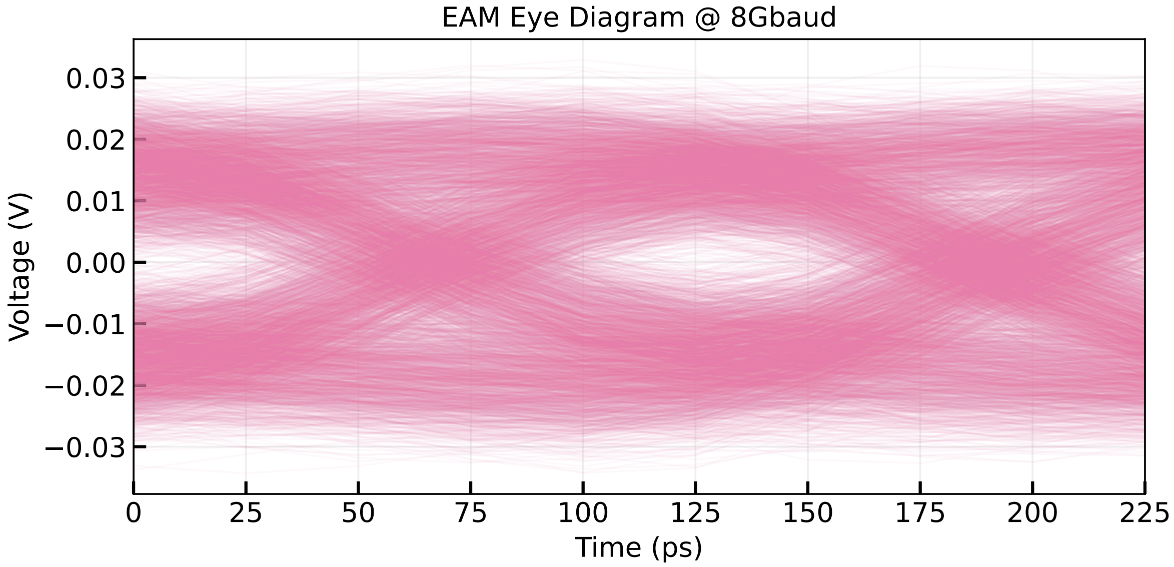

Supplementary Figure 6: EAM Eye Diagram at 8 GBaud Symbol rate

Supplementary Note 6. PCB Design

A carrier PCB was designed to establish an electrical connection to the chip. The chip is glued to the bare copper area in the center and connected to the PCB via bond wires. The input modulation voltages from the RF-DACs enter the board differentially via Samtec ARC6 connectors and are transitioned to single-ended signals using local baluns (Mini-Circuits' TCM2-43X+). All input modulators share a common connection to the bias voltage, which is decoupled with capacitors located directly underneath. The photodiode output currents are routed to SMA connectors at the left edge to be connected to external TIAs. The TIA inputs provide a 50R path to ground, while the other end of the photodiodes shares a common, locally decoupled bias voltage like the input EAMs. The TIA outputs are connected to a second set of SMA connectors, transitioned to differential signals using the same baluns as for the inputs and

then routed to ARC6 connectors to be connected to the RF-ADCs. Although not high-speed, the bias and weight voltages use the same type of connector. All traces are impedance-matched to 50R (single-ended) or 100R (differential) and groupwise length-matched.

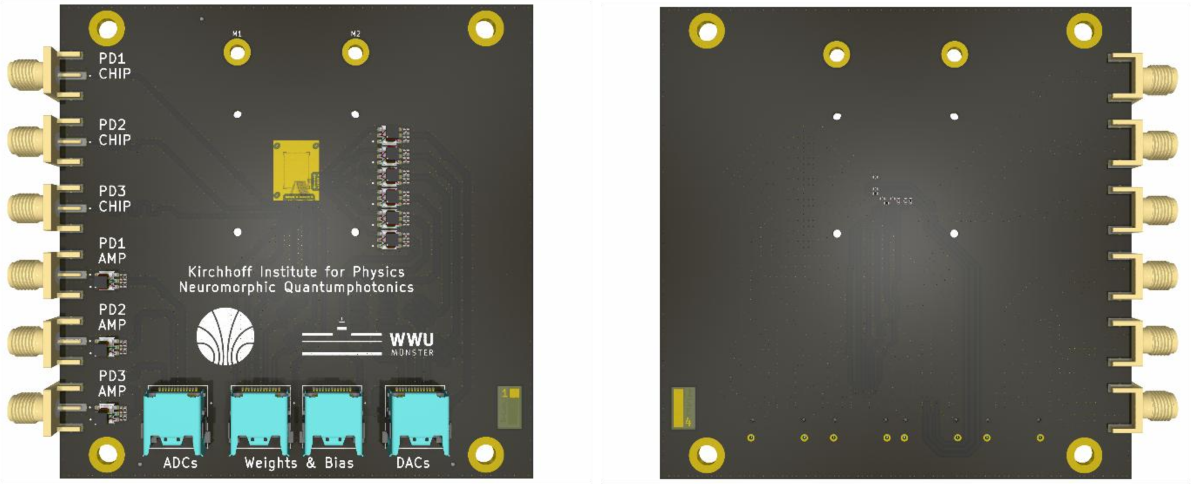

Supplementary Figure 7: Printed Circuit Board for electronic interfacing

Supplementary Note 7. Input Encoding

We are using a zero-mean, four-sample, return-to-zero, alternating encoding, such that the TIA's see only changes around the optical bias. That lets us represent signed values even though the modulator only attenuates power.

Our desired input symbol is

$$k_n \in [-1,1]$$

We encode it into 4 DAC samples without a DC part, setting the operation / symbol rate of the system to a quarter of the DACs speed:

$$\boldsymbol{s_n} = [s_{4n+1}, s_{4n+2}, s_{4n+3}, s_{4n+4}] = [k_n, 0, -k_n, 0]$$

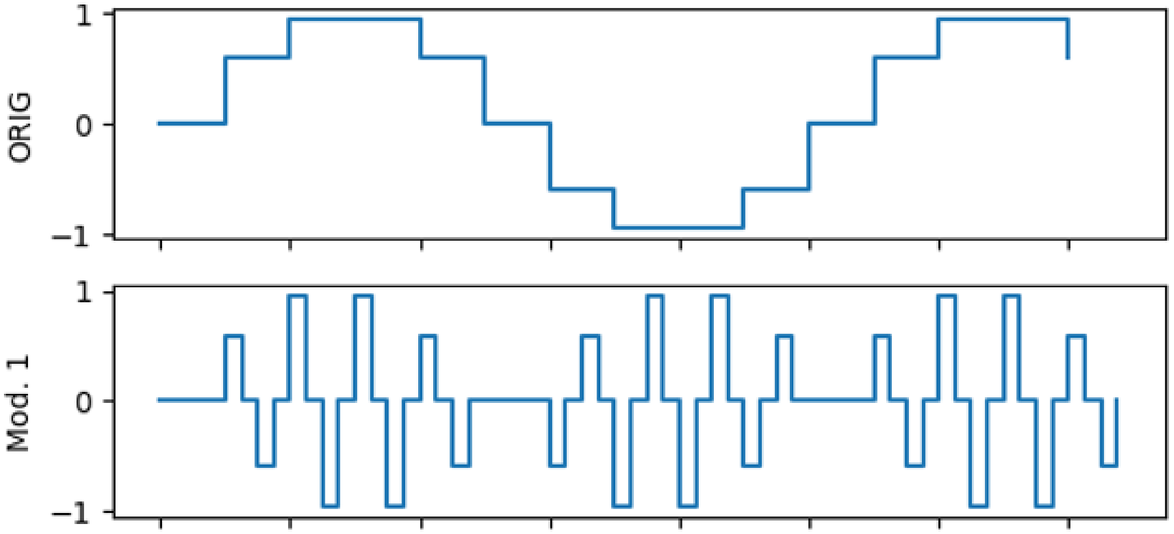

Supplementary Figure 8: Modulation Schemes. A single symbol is encoded into four samples. We send 4 samples per symbol, two samples with the value of the symbol but with flipped signs and insert zeros in between

We bias the EAM at V_b and scale the DAC swing by V_p, applying the voltage v_m at the modulator

$$v_m = V_b + V_p * s_m$$

Assuming linear transmission around the bias point

$$T(v_m) \approx T_b + \alpha V_p s_m$$

With $\alpha = \frac{dT}{dV}|V_B$.

The photocurrent is

$$i_m = RP_{in}T(v_m)$$

With the photodiode responsivity R and the input optical power P_{in}. The output after the AC-coupled TIA is

$$y_m = GRP_{in}\alpha V_p s_m = C * s_m$$

With the gain of the TIA *G.*
We drive the ADC at half the speed of the DAC and sample at the non-zero time slots:

$$\tilde{k}_n = (\mathrm{y}_{4\mathrm{n}+1} - \mathrm{y}_{4\mathrm{n}+3}) = C(s_{4n+1} - s_{4n+3})$$

The two nonzero samples produce opposite-signed fluctuations around the bias, so the AC-coupled output carries a signed value even though the optical power itself is non-negative.

Normalizing with $\frac{1}{2C}$ recovers the encoded symbol

$$\frac{1}{2C} * \tilde{k}_n = \frac{1}{2C} * C(s_{4n+1} - s_{4n+3}) = \frac{1}{2C} * C\big(k_n - (-k_n)\big) = k_n$$

Supplementary Note 8. Balanced Weight Encoding

Balanced encoding uses one row as a reference. The FPGA subtracts the reference transmission from the main transmission, so the difference is a signed value. Let $\mathrm{w_p}^{\mathrm{max}}$ and $\mathrm{w_p}^{\mathrm{min}}$ be the physical weights at maximum and minimum transmission. Define the neutral level

$$w_p^{ref} = \left(w_p^{max} + w_p^{min}\right)/2$$

and the span

$$\Delta w_p = w_p^{max} - w_p^{min}$$

two physical weights w_{p1}, w_{p2} encode a target weight w in [−1, 1]

$$w = \left(w_{p1} - w_{p2}\right)/\Delta w_p,$$

$$w_{p1} = \mathrm{w_p^{ref}} + \left(\Delta \mathrm{w_p}/2\right) * \mathrm{w},$$

$$w_{p2} = \mathrm{w_p^{ref}} - \left(\Delta \mathrm{w_p}/2\right) * \mathrm{w}$$

We symmetrically offset the reference row around w_p^{ref}, so the main minus reference photocurrent spans $[-\Delta w_p, \Delta w_p]$

Weight and Crosstalk Calibration

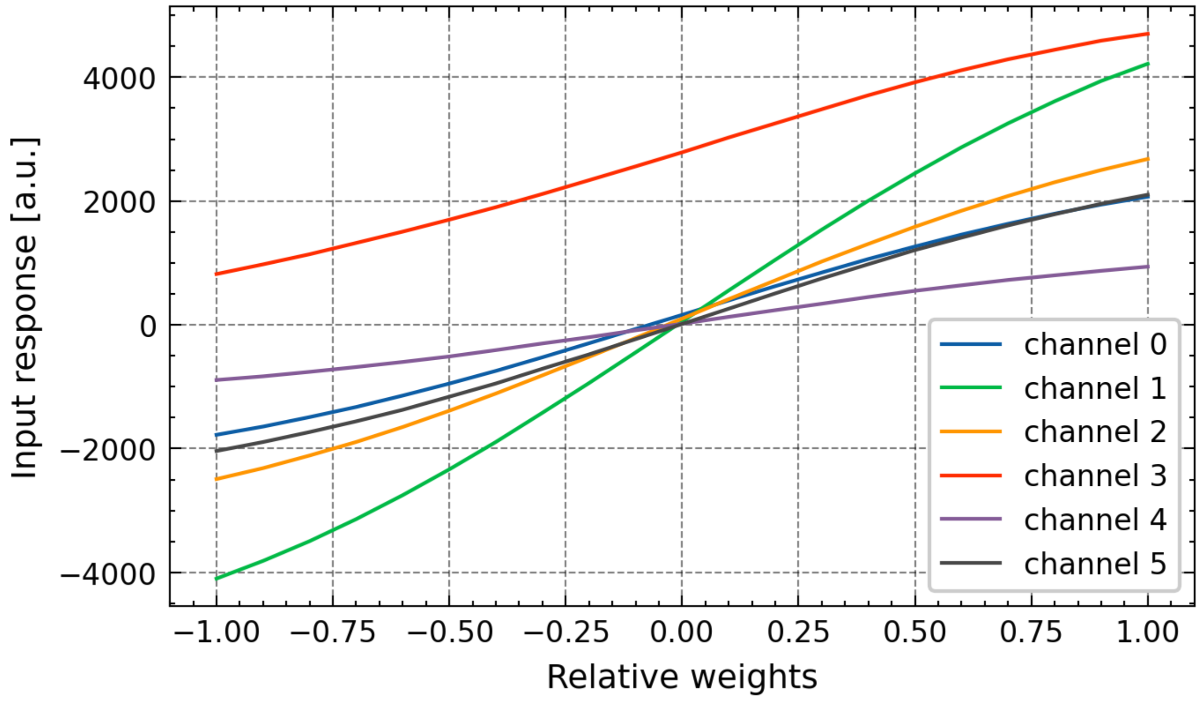

Supplementary Figure 9: Typical weight map. The measurement captures the input responses of each channel for the whole range of relative weights. The nonlinear scaling becomes obvious, resembling the EAM transmission curve as well as the strongly inhomogeneous behaviour between different channels. The red curve indicates a channel where the reference row in the crossbar array is not working; therefore, only positive weights can be set with that specific channel.

Weight-map calibration:

For each channel c, we sweep the relative range $r \in [-1,1]$ while holding other channels at zero and record the input response y.

$$\vec{X} * \vec{w} = 1 * \vec{e}_{ctest} * \sum_i w_i * \vec{e}_i = w_{ctest}$$

This yields a per-channel calibration curve f_c: $r \mapsto y$, sampled on a fixed grid r_j and stored as a matrix $m_{c,j}=f_c(r_j)$. Importantly, f_c implicitly includes the effects of microcomb line power non-

uniformity and the wavelength-dependent EAM response, since both directly determine the measured optical output for a given drive. The common realizable target range is the intersection of per-channel ranges $[\min_j m_{c,j}, \max_j m_{c,j}]$.

Weight setting:

Given desired $\boldsymbol{w}$, we linearly map $\boldsymbol{w}$ into target units $\boldsymbol{t}$ inside the common feasible range, then obtain the required drives by inverting each channel's calibration curve via interpolation, $\rho_c = f_c^{-1}(t_c)$. When available, a measured linear crosstalk matrix C is applied in a single pass as $t_c \leftarrow t_c - \sum_k C_{c,k} \rho_k$ before reinversion.

Crosstalk analysis and correction:

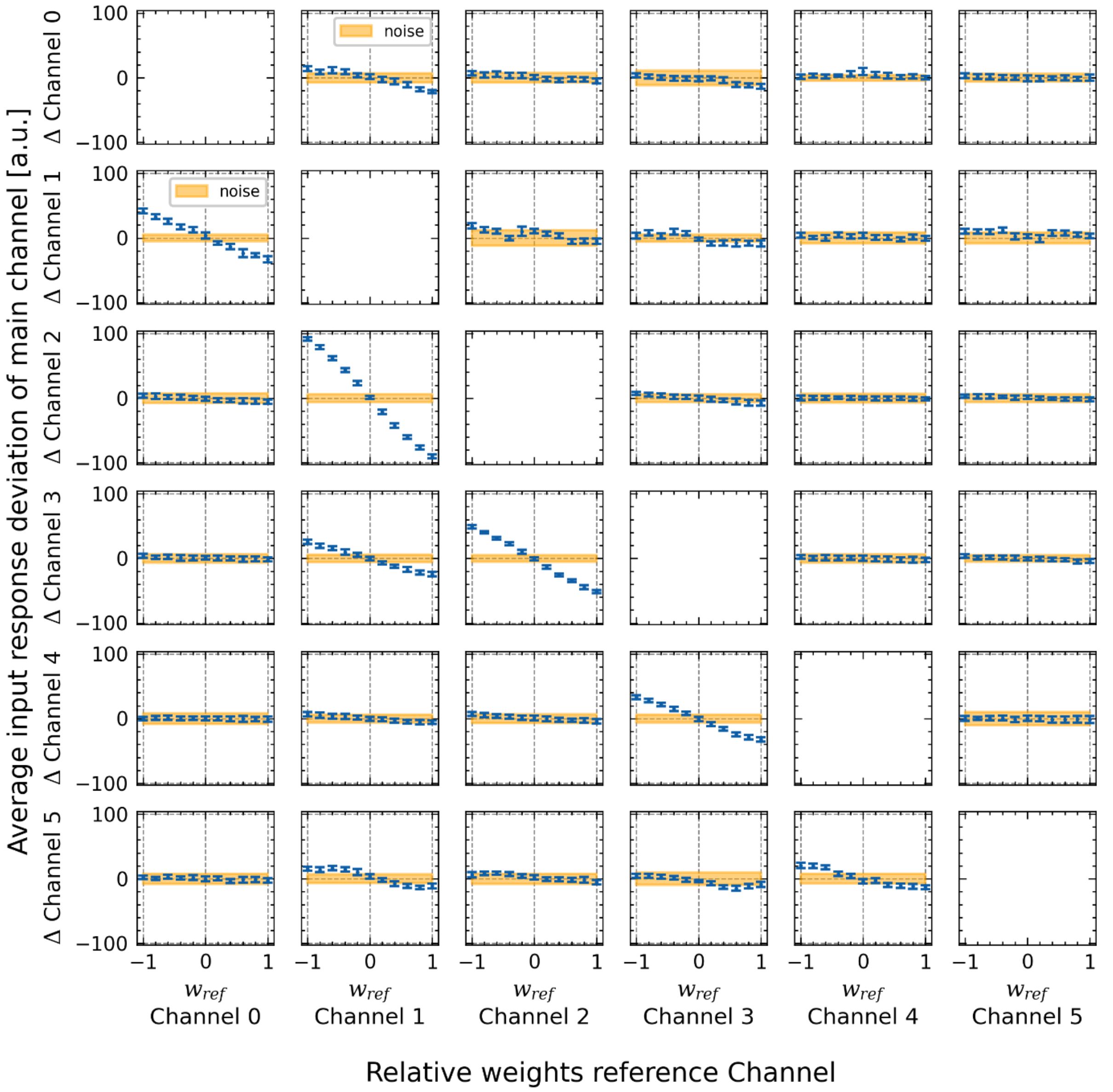

Supplementary Figure 10: Typical crosstalk measurement. The x-axis shows the relative weights of the respective reference channel, and the y-axis depicts the average deviation of the weight map in units of the output response for the respective main channels. The orange region indicates deviations within the weight mapping that can be explained just by the ground noise of the system.

Full multidimensional mapping is infeasible, so correlations are measured pairwise. For each test channel, its map is remeasured while stepping one reference channel through 11 values with all others at zero, and this is repeated so every channel serves once as the reference. Linear fits without intercept provide a slope for each test–reference pair, which is then used as a linear

correction when choosing target weights. The strongest couplings are between physically adjacent connectors. For larger arrays, it is efficient to measure only neighboring pairs.

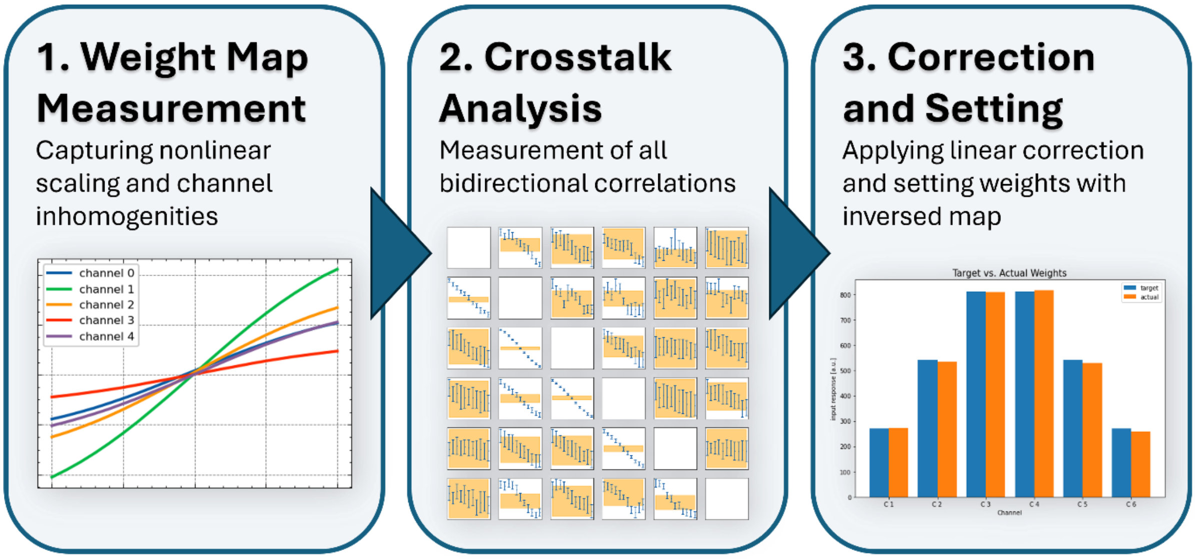

Supplementary Figure 11: Flowchart of the weight mapping

Supplementary Note 9. Efficiency Analysis

To calculate the system efficiency, we consider the following contributions and estimate both current values as well as theoretical limits. As the power consumption of the EAMs and photodiodes depends on the incident optical power as well their bias voltage, we make the following assumptions:

- The combined optical input power at all channels equals P_{in}.
- The input modulators are biased at -2 V, where approximately half of the input power is absorbed.
- On average, 50 % of the remaining optical power is absorbed by the weights which are biased in the range [0 V; -4 V] with an average bias voltage of -2 V.

- The combined optical output power that reaches the photodiodes equals $P_{in} \cdot 0.5 \cdot 0.5 \cdot \frac{1}{n}$, incorporating the input modulator IL, the weight IL and directional coupler loss. The photodiodes are biased at -3 V.

1) Optical input power (P_{opt}):

   The total optical input power to our system was $P_{in} = 27\ mW$.

   The frequency comb was pumped with ca. 700 mW of electrical input power and generated approximately 10 mW of usable optical power. Another 300 mW is assumed for heater control power. The efficiency of the subsequent amplifiers is not known but we assume a conservative 10 % wall-plug efficiency. This results in

$$P_{opt} = 700\text{ mW} + 300\text{ mW} + (27\ mW - 10\text{ mW}) * 10 = 1170mW$$

   With current soliton microcomb and amplifier technology, we expect a possible wall-plug effiency for the whole optical chain of 10 % which would result in a potential power consumption of

$$\widehat{P_{opt}} = 10 \cdot P_{in} = 270\ mW$$

2) Input modulator bias (P_{imb}):

   At the bias point, half of the photons are absorbed by the input modulators, resulting in a photocurrent of $\frac{q \cdot 0.5 \cdot P_{in}}{E_{ph}} \cdot \eta_{ext,eam}$ which requires an electrical bias power of $P_{imb} = \frac{q * 0.5 \cdot P_{in}}{E_{ph}} \cdot \eta_{ext,eam} \cdot 2$ V. Assuming an external quantum efficiency of $\eta_{ext,eam} = 0.8$, this results in $P_{imb} = 27\ mW$. This is a theoretical limit when using EAMs.

3) Input modulator drive (P_{dac}):

The input EAMs are directly driven by the DACs without additional amplification. The input modulation is therefore given by the power consumption of the DACs driving capacitive loads (or resistive loads in case of termination). We estimate the DAC power using the Xilinx Power Design Manager (PDM) as ca. 300 mW per channel, i.e.

$$P_{dac} = 9 \cdot 300 \text{ mW} = 2700 \text{ mW}$$

To estimate a theoretical potential for the DACs, the 300 mW per channel is split into two parts. About 100 mW are required to electrically drive the output. It is assumed that the remaining 200 mW scale similary to the ADCs when going to lower precision (see section for P_{adc}) which results in

$$\widehat{P_{dac}} = 9 \cdot \left(100 \text{ mW} + \frac{200 \text{ mW}}{64}\right) \approx 900\, mW$$

4) Weight modulation (P_w):

Weights are assumed to be quasi-static. We therefore only consider the power required to set their operating point, similar to the bias power of the input modulators. Assuming that the absorption current scales linearly with the applied bias voltage, that full absorption is achieved at -4 V and that all weights are uniformely distributed within the interval [0 V; -4 V], the estimated weight bias power is:

$$P_w = \int_{0V}^{4V} \frac{q*0.5 \cdot P_{in}}{E_{ph}} \cdot \eta_{ext} \cdot v^2 \cdot \frac{1}{4\,V} dv = \frac{q*0.5 \cdot P_{in}}{E_{ph}} \cdot \eta_{ext} \cdot \frac{1}{4\,V} \cdot \int_{0V}^{4V} v^2\, dv = \frac{q*0.5 \cdot P_{in}}{E_{ph}} \cdot \eta_{ext} \cdot \frac{1}{4\,V} \cdot \frac{64}{3} = 72\, mW$$

This is a fundamental limit for EAMs and cannot be reduced further.

5) Photodiode bias (P_{pd}):

The electrical power required for biasing the photodiodes equals the photocurrent times the bias voltage which results in

$$P_{pd} = q \cdot P_{in} \cdot 0.5 \cdot 0.5 \cdot \frac{1}{n} \cdot \eta_{ext,pd} \cdot 3\ \text{V} = 6.75\ mW$$

This is a fundamental limit for PDs and can only be reduced by lowering the bias voltage, resulting in a lower bandwidth and lower gain.

6) TIA (P_{tia}):

We used commercially available lab TIAs (FEMTO, HSA-Y-1-60) with approximately 3000 mW power consumption per channel i.e.

$$P_{tia} = 3 \cdot 3000\ \text{mW} = 9000\ \text{mW}$$

Estimating a potential efficency for TIAs is difficult as most power in current high-speed amplifiers is dissipated in the bias circuitry but it is assumed that the required TIA power equals at least the minimum DAC drive power, i.e.

$$\widehat{P_{tia}} = 3 \cdot 100\ \text{mW} = 300\ \text{mW}$$

7) ADC (P_{adc}):

The ADC power was estimated by the AMD PDM as approximately 270 mW per channel (at the actual operating conditions). Therefore

$$P_{adc} = 3 \cdot 270\ \text{mW} = 810\ \text{mW}$$

While the used ADCs are highly optimized and best-in-class, they are unnecessarily precise. Assuming the Walden FOM for ADCs, the required energy per conversion doubles with every additional bit of precision [11]. Reducing the precision to 8-bit would therefore reduce the power by a factor 64.

$$\widehat{P_{adc}} = 3 \cdot 270 \text{ mW} \cdot \frac{1}{64} = 13 \text{ mW}$$

8) Electronic control (P_{ec}):

The AMD PDM estimates a power consumption of approximately 4 W for the implemented RFSoC design (excluding the RF DACs and ADCs), with ca. 2 W attributed to both the Processing System and the Programmable Logic.

$$P_{ec} = 4000 \text{ mW}$$

Removing the high-level control PS and using a dedicated ASIC for processing the digital data streams, we estimate that the required control power can be reduced to

$$\widehat{P_{ec}} = 1000 \text{ mW}$$

Total:

Actual Consumption:

$$P_{tot} = P_{opt} + P_{imb} + P_{dac} + P_w + P_{pd} + P_{tia} + P_{adc} + P_{ec}$$

$$= 1170\, mW + 27\, mW + 2700 \text{ mW} + 72\, mW + 6.75\, mW + 9000 \text{ mW}$$

$$+ 810 \text{ mW} + 4000 \text{ mW} = \ 17.78\, W$$

Projected Consumption:

$$\widehat{P_{tot}} = \widehat{P_{opt}} + P_{imb} + \widehat{P_{dac}} + P_w + P_{pd} + \widehat{P_{tia}} + \widehat{P_{adc}} + \widehat{P_{ec}}$$

$$= 270mW + 27mW + 900 \text{ mW} + 72mW + 6.75\, mW + 300 \text{ mW} + 13 \text{ mW}$$

$$+ 1000 \text{ mW} = \ 2.5\, W$$

Supplementary References

[1]. T. J. Kippenberg *et al.*,Dissipative Kerr solitons in optical microresonators. *Science* 361,(2018).

[2]. Stern, B., Ji, X., Okawachi, Y. *et al.* Battery-operated integrated frequency comb generator. *Nature* 562, 401–405 (2018)

[3]. Raja, A.S., Voloshin, A.S., Guo, H. *et al.* Electrically pumped photonic integrated soliton microcomb. *Nat Commun* 10, 680 (2019).
[4]. Shen, B., Chang, L., Liu, J. *et al.* Integrated turnkey soliton microcombs. *Nature* 582, 365–369 (2020).
[5]. Voloshin, A.S., Kondratiev, N.M., Lihachev, G.V. *et al.* Dynamics of soliton self-injection locking in optical microresonators. *Nat Commun* 12, 235 (2021).
[6]. Chao Xiang *et al.*, Laser soliton microcombs heterogeneously integrated on silicon. *Science* 373, 99-103(2021).
[7]. Oraevsky A N, Yarovitsky A V and Velichansky V L 2001 *Quant. El.* 31 897
[8]. N. M. Kondratiev, V. E. Lobanov, A. V. Cherenkov, A. S. Voloshin, N. G. Pavlov, S. Koptyaev, and M. L. Gorodetsky, "Self-injection locking of a laser diode to a high-Q WGM microresonator," Opt. Express 25, 28167-28178 (2017)
[9]. N. M. Kondratiev, V. E. Lobanov, E. A. Lonshakov, N. Yu. Dmitriev, A. S. Voloshin, and I. A. Bilenko, "Numerical study of solitonic pulse generation in the self-injection locking regime at normal and anomalous group velocity dispersion," Opt. Express 28, 38892-38906 (2020)
[10]. G. Huang, E. Lucas, J. Liu, A. S. Raja, G. Lihachev, M. L. Gorodetsky, N. J. Engelsen, and T. J. Kippenberg, "Thermorefractive noise in silicon-nitride microresonators. " *Physical Review A*, *99*(6), 061801, (2019).
[11]. Murmann, B. The Race for the Extra Decibel: A Brief Review of Current ADC Performance Trajectories. *IEEE Solid-State Circuits Mag.* **7**, 58–66 (2015).
